# Acquisition of concrete and abstract words is modulated by tDCS of Wernicke’s area

**DOI:** 10.1038/s41598-020-79967-8

**Published:** 2021-01-15

**Authors:** Diana Kurmakaeva, Evgeny Blagovechtchenski, Daria Gnedykh, Nadezhda Mkrtychian, Svetlana Kostromina, Yury Shtyrov

**Affiliations:** 1 Laboratory of Behavioural Neurodynamics, Saint Petersburg University, Saint Petersburg, 199004 Russian Federation; 2 Center of Functionally Integrative Neuroscience (CFIN), Department of Clinical Medicine, Aarhus University, 8000 Aarhus, Denmark

**Keywords:** Cognitive neuroscience, Learning and memory

## Abstract

Previous behavioural and neuroimaging research suggested distinct cortical systems involved in processing abstract and concrete semantics; however, there is a dearth of causal evidence to support this. To address this, we applied anodal, cathodal, or sham (placebo) tDCS over Wernicke’s area before a session of contextual learning of novel concrete and abstract words (n = 10 each), presented five times in short stories. Learning effects were assessed at lexical and semantic levels immediately after the training and, to attest any consolidation effects of overnight sleep, on the next day. We observed successful learning of all items immediately after the session, with decreased performance in Day 2 assessment. Importantly, the results differed between stimulation conditions and tasks. Whereas the accuracy of semantic judgement for abstract words was significantly lower in the sham and anodal groups on Day 2 vs. Day 1, no significant performance drop was observed in the cathodal group. Similarly, the cathodal group showed no significant overnight performance reduction in the free recall task for either of the stimuli, unlike the other two groups. Furthermore, between-group analysis showed an overall better performance of both tDCS groups over the sham group, particularly expressed for abstract semantics and cathodal stimulation. In sum, the results suggest overlapping but diverging brain mechanisms for concrete and abstract semantics and indicate a larger degree of involvement of core language areas in storing abstract knowledge. Furthermore, they demonstrate a possiblity to improve learning outcomes using neuromodulatory techniques.

## Introduction

Most of the available behavioural and neuroimaging studies suggest distinct cognitive mechanisms underpinning encoding and storage of abstract and concrete semantics. At conceptual level, as often pointed out^1^, concrete concepts are characterised by clear references to material, physical objects (e.g. *cat, car*), whereas references of abstract concepts are not physical entities, but more complex mental states (e.g. *dream, sadness*), conditions (*uncertainty*), relationships (*friendship*) or situations (*encounter*). At behavioural level, these distinctions are known to be most typically reflected in the so-called “concreteness effect”^2,3^. As demonstrated in a number of experiments, which used various behavioural tasks (such as word recognition, naming, lexical decision etc.), concrete concepts are, as opposed to abstract ones, better remembered^4^, recognized^5^, faster read and comprehended^6^, and faster learnt^7^. Numerous attempts at explaining these differences have been made, enlisting various approaches in different fields, from purely theoretical linguistic and cognitive frameworks to neuroimaging experiments. Various theoretical approaches place emphasis on different features of concept perception; among the most influential are dual coding^8^, context availability^6^, hierarchical and associative information processing^9^ theories, and some others^10^.

Investigation of differences between the processing of abstract and concrete semantics is of interest not only for theoretical studies of language but also for understanding how complex cognitive phenomena are implemented at the neural level. Neuroimaging data appear to indicate that the processing of concrete and abstract words involves different brain areas, at least in part, as demonstrated in EEG^11,12^ and fMRI^13–15^ studies supporting the extended dual-coding theory^11^. The available neuroscience-based theoretical accounts suggest that, whereas concrete word representations are more distributed and involve, depending on the exact semantic reference, different modality-specific areas, the abstract semantics relies more on the core language areas of the left hemisphere, i.e. temporo-parietal and inferior-frontal neocortex^3,5,16^. However, brain activation patterns as such do not necessarily indicate that a particular area is necessary for coding certain information, and the differences in activations may be due to secondary phenomena rather than the key underlying mechanisms^17^. This would require methods that can help establish causality. Neurostimulation techniques (such as transcranial magnetic or direct-current stimulation, TMS/tDCS) may provide a possibility to assess causally the involvement of particular brain regions in specific cognitive functions. At this time, there is a dearth of TMS/tDCS experiments addressing the concrete-abstract semantic distinction. Furthermore, even existing studies have not been always able to show any differential influence of stimulation on concrete and abstract word processing. For instance, it was shown that anodal tDCS over dorsolateral prefrontal cortex (DLPFC) or parietal cortex facilitated the retrieval of both semantic types equally^18^. On the other hand, Vukovic et al.^19^, using TMS, suggested prefrontal and premotor areas to take part in abstract word comprehension; that study, however, did not explore areas outside the frontal lobe, being mainly focused on action semantics. Similarly, other TMS studies have shown that the processing of abstract and concrete phrases differentially modulates motor cortex and cortico-spinal excitability^20,21^. Neither of these have addressed the core language systems. This has recently been done using direct electrical stimulation (DES) during awake surgery, showing that DES applied over the left BA44, which is a part of Broca’s area and inferior frontal gyrus (IFG), decreases the accuracy of semantic judgement task for abstract words, whereas for concrete words similar results were observed when left BA38 in the temporal lobe was stimulated^22^. This is an important finding, although one has to be cautious generalising results obtained surgically in neurological patients to healthy population.

Another important issue is that, when evaluating the mechanisms responsible for different semantic processes, most studies deal with pre-existing representations of native words. When, however, existing concrete and abstract words are compared, the results are confounded by their physical (e.g. duration), psycholinguistic (frequency), phonological/orthographic properties, and individual learning trajectories. For instance, abstract nouns in English are typically longer than concrete ones^23^. Could it be this physical, rather than semantic, difference that affects the acquisition and processing of abstract and concrete semantic, e.g. penalising longer words? As abstract words are typically acquired later in life^24^, would any differences in behavioural and neural measures reflect their semantics per se or rather this diverging acquisition background? Similarly, as concrete words are used more frequently than abstract ones^25,26^, would this lexical frequency difference confound any comparison between them (but note also an alternative account of frequency norms in English^23,27^)? In our view, one way to circumvent these difficulties and compare mechanisms for concrete and abstract word comprehension without confounds would be to study the formation of representations for new words in a controlled environment, balancing them for the overall physical and psycholinguistic features as well as ensuring a similar acquisition mode. This is what we aimed at in the current study.

Effects of non-invasive brain stimulation on learning new words have been demonstrated in healthy individuals and for language re-acquisition after stroke-induced aphasia^28–30^. For instance, tDCS has been shown to enhance language learning performance^31–33^. A recent neurostimulation study^34^ revealed that anodal tDCS over Wernicke's area in a word learning task significantly improved the accuracy and decreased latencies in a picture-naming task, while another experiment^28^ showed faster and better associative verbal learning with the anodal tDCS over the posterior part of the left perisylvian area, compared to sham (placebo) condition.

However, no non-invasive stimulation study has so far directly compared the learning of concrete and abstract semantics. A direct comparison of novel concrete vs. abstract word learning after tDCS of different polarities was the aim of the present experiment.

Critically, the available accounts claim that, whereas representations of concrete words are distributed across different areas of the two hemispheres and involve modality-specific areas, the abstract semantics is predominantly represented by the core language systems of the left hemisphere, which, in turn, suggests stronger impact of their stimulation on abstract word acquisition. This prediction, in turn, points to two main potential targets for such stimulation: Broca’s area in the left inferio-frontal cortex or temporo-parietal cortices in Wernicke’s area and its vicinity. In this first study on this topic, we chose Wernicke’s area, a key language area in the neocortex, linked with the coding, storage and comprehension of various types of semantics^35^, which has already been successfully modulated in word-learning experiments^28,34^. One reason for this choice was to avoid interference with language production mediated by Broca's area and required in assessing the outcomes of the learning task. We expected Wernicke’s area tDCS to affect the acquisition of concrete and abstract semantics, with a stronger influence for the latter.

We used anodal and cathodal stimulation in different groups of subjects, with a further control group subjected to sham stimulation, which imitates tDCS set-up without delivering actual current to the participant’s head. Diverging effects of tDCS polarity are known from a large number of motor-system studies^36–38^, which demonstrated the excitatory effect of anodal versus the inhibitory effect of cathodal stimulation. However, studies addressing other areas and higher cognitive functions have largely failed to confirm this dichotomy. Whereas those studies that applied anodal tDCS mostly did find facilitatory effects^28,39,40^, for cathodal stimulation the results diverge across the literature. Some of the investigations dedicated to cognitive functions showed an inhibitory effect of cathodal stimulation^41,42^, whereas others showed performance improvement after cathodal tDCS^43,44^. Moreover, a recent tDCS study of language processing targeting Wernicke’s area demonstrated that both anodal and cathodal stimulation improved semantic processing in the lexical decision task in comparison with the sham condition, suggesting that cathodal inhibition, common for motor studies, can not be simply assumed to apply to the language function in the same way^45^. This effect can also be explained in terms of the hypothesis that the influence of tDCS makes the neural system sensitive to other stimuli by shifting the excitation thresholds up or down, rather than causes inhibition and excitation per se^33,46–48^. That makes exact predictions for the inhibitory and facilitatory effects of cathodal and anodal stimulation difficult and instead warrants experimental investigation of both polarities, which is what we have attempted in the present study.

As stimuli, we created a set of novel concrete and abstract concepts, not known to our subjects previously. Furthermore, we fully controlled the surface features of the stimuli and counterbalanced the use of different word forms for encoding concrete or abstract semantics by rotating them across participants. To mimic natural acquisition of new words in real life, we presented them in the context of short stories, enabling our participants to infer the meaning of novel semantics through this naturalistic (yet fully controlled) context. Finally, we used a comprehensive set of tasks to assess the acquisition of new words at both lexical and semantic levels. To account for both immediate effects of learning and overnight consolidation^49^, we tested the behavioural outcomes following the learning session and 24 h later, after an overnight sleep. We expected that (1) in line with previous research, tDCS would affect the efficiency of learning, (2) these effects may diverge between the anodal and cathodal stimulation, as can be expected based on previous research suggesting suppression after cathodal and facilitation after anodal tDCS^36,37,50^ (but see the discussion of cathodal effects above), (3) the stimulation would affect abstract and concrete word acquisition to different degrees, as suggested by previous neuroimaging evidence indicating differential activation of temporo-parietal structures in the processing of these word types^51^.

## Methods

### Participants

Right-handed (handedness assessed using an abridged version of the Edinburgh Handedness Inventory^52^) native monolingual Russian speakers with normal or corrected to normal vision, no history of neuropsychiatric disorders, trauma or drug abuse participated in the study. The sample included 72 participants randomly assigned to three groups (N = 24, 4 male, 20 female in each, 17–35 y.o.). The three groups did not differ statistically in their age (p = 0.231, χ^2^(2) = 2.933), handedness (p = 0.528, χ^2^(2) = 1.276), age of the first experience of foreign language acquisition (p = 0.331, χ^2^(2) = 2.210) and the level of education (measured in years; p = 0.068, χ^2^(2) = 5,378) as tested using Kruskal–Wallis one-way analysis of variance. The study protocol was approved by the St. Petersburg University Ethics Committee; all experiments were performed in accordance with the relevant guidelines and regulations and all participants gave their written informed consent to take part in the experiments.

### Materials

New, previously unfamiliar word forms (“pseudowords”) were developed based on real words of the Russian language by changing their final syllables (e.g. гapдepoб → гapдeнaл* [garderobe → gardenal*], i.e. *wardrobe* → *novel pseudoword*). These pseudowords resembled existing words in terms of orthographic and phonological structure such that participants would not perceive them as words of a foreign language which typically differ in phonetics from native words. In total, 30 words (3 sets of 10) were chosen as a base for the experimental stimulus set to be modified via recombination to create novel pseudowords. These were assigned to abstract or concrete novel word categories and presented across the subject groups in a counterbalanced fashion to avoid any random effects of surface stimulus properties (see also Blagovechtchenski et al., for a more detailed methods description^33)^. The base words all had eight letters/phonemes and consisted of three syllables with CVC–CV–CVC structure (where C stands for consonant and V for vowel). These lists were chosen such that they did not differ statistically on their lemma and the ultimate syllable (trigram) frequencies, according to the Russian National Corpus (RNC) psycholinguistic database (http://www.ruscorpora.ru/), to prevent any frequency related confounds. Based on these words, new pseudowords were created using the following procedure: the first two syllables (CVC-CV) of the word were preserved and the ultimate CVC segment was changed (by rotating these syllables across lists) to create novel items not existing in Russian (e.g., мaндapин, кapдинaл → кapдиpин*, мaндaнaл* [mandarin, cardinal → cardirin*, mandanal*]). Apart from novel word forms, novel semantics was created to be learnt as their meaning. For novel concrete words, it was developed using rare, obsolete or ancient objects, which were unfamiliar to the participants (e.g. *a medieval contraption for catching fleas in one’s hair or wig*). New abstract concepts were adopted from foreign cultures, with the requirement that no words for these concepts existed in the participants’ native language (e.g. *the feeling of having too many passwords, not being able to remember all of them*). In total, 10 novel concrete and 10 novel abstract concepts were created, to be paired with new word forms in a contextual learning setting. None of these were known to the participants, as established in a separate survey. Both sets were evaluated by participants in terms of concreteness/abstractness, emotional valency, and imageability on a 7-point Lickert scale after the experiment. Significant differences between them were found for concreteness/abstractness as expected, confirming the validity of the developed stimulus set.

**Figure 1:**
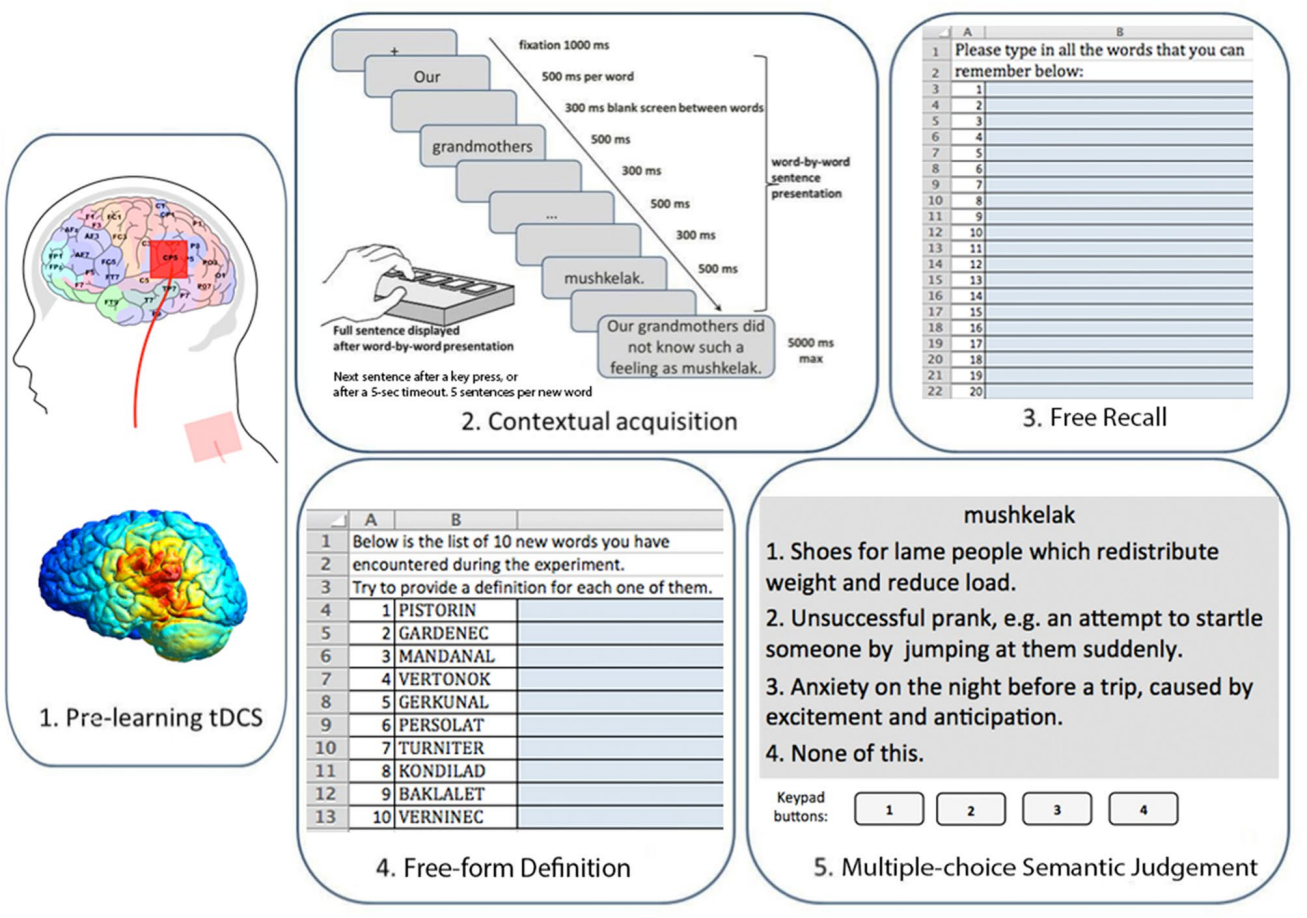
Diagram of main experimental procedures. (Note that stimulus translation from Russian into English is approximate, see Methods for details).

Each novel concept was described in a set of five sentences presented visually (see examples of contextual sentences in Fig. 1 and in Supplementary materials, Table 1). Thus, 100 contextual sentences were developed in total. Each sentence consisted of eight words. Experimental pseudowords always took place at the end of sentences and were presented in the nominative or accusative case (which share the same surface form in Russian, not requiring inflectional endings), such that their surface form was not inflected differently in different sentences.

### Procedure

Experimental procedure consisted of five steps, illustrated in Fig. 1 and described below.

The experiment was conducted in an electrically shielded and soundproof chamber (Neuroiconica, St. Petersburg, Russia). A battery-driven stimulator (BrainStim SYS, E.M.S, Bologna, Italy) was used. Direct current of low intensity (1.5 mA) was delivered to the subjects’ skin through a pair of rubber electrodes covered with electrically conductive Geltek-Medica gel. The active electrode (5 × 5 cm) was placed on the scalp over Wernicke's area (CP5 electrode location according to the extended international EEG 10–20 system); the reference electrode (5 × 10 cm) was located at the base on the left side of the neck^33^. The chosen electrode sizes, current density, and duration of the stimulation were standard and optimal for producing sufficiently local stimulation^53,54^. Since tDCS is not very focal with respect to its neuroanatomical precision, not only the classical regions related to the Wernicke’s area may be stimulated with this particular montage, but also some nearby regions. However, according to the model of the current flow, the main effects of the present stimulation here should still be associated with the temporo-parietal cortex within Wernicke’s area, where the current intensity is highest. In Fig. 1 (left lower) we show the simulation model of the current spread for the current montage and stimulation settings, built using SimNIBS v. 3.1.2 software (https://simnibs.github.io/simnibs/build/html/index.html; ^55);^ note that neuroanatomically the model is identical for both stimulation polarities, only the current direction is different.

The three groups of participants received sham (placebo), cathodal, or anodal stimulation while seating with open eyes without any distracting conditions. The stimulation procedure lasted for 15 min, including a 30-s ramp-up and a 30-s ramp-down periods. The sham stimulation procedure corresponded to the one described above except that the current was applied only briefly (30 s) at the beginning and the end of the session: during the first and last 30 s of the 15-min tDCS session, an electric pulse of a triangular shape with a maximum of 1.5 mA was applied. The main sensation of stimulation is associated with changes in current intensity^56^. In placebo stimulation condition, the subjects are exposed to the same changes in current strength as in real anodal or cathodal conditions, but the phase of long-term exposure to current is excluded. Thus, the sham session participants had a generally similar experience to that of real tDCS, which normally amounted to a painless tingling sensation during the initial and final seconds of the stimulation session.

Immediately after the tDCS application, the participants underwent contextual acquisition of novel words. They were seated in front of a computer monitor and instructed to read stimulus sentences carefully. Each sentence started with a word-by-word presentation, followed by the presentation of the entire sentence on the screen to ensure full understanding. Participants had to press the spacebar with their index finger of the left hand after reading the complete sentence. The duration of the sentence presentation was 5000 ms. Then a fixation cross (“+”) appeared in the center of the screen for 1000 ms and the presentation of the next sentence began. The sets of sentences were separated from each other by a triple crosshair (“+++”) presented for 2000 ms and another fixation cross displayed for 1000 ms. Each word was presented for 500 ms following a 300-ms empty screen between words within one sentence. A short training block was presented prior to the main part of the experiment. The main experimental block was divided into two sub-blocks, with a short break between them to reduce fatigue. The average duration of the acquisition phase was 25 min. The contextual acquisition part of the experiment was implemented in NBS Presentation 20.0. The background colour was grey (RGB: 125, 125, 125), the text colour was black (RGB: 0; 0; 0), font—Arial, font size—24.

Stimulation of language cortices may produce a complex pattern of behavioural outcomes, therefore a battery of different tests was used to assess the acquisition of novel word forms on both lexical and semantic levels (1) immediately after learning and, (2) to allow for consolidation period, on the following day. For these tests, the stimulus word forms were split into two subsets to be assessed on Day 1 and Day 2, to avoid carry-over of stimulus exposure during Day 1 assessment into Day 2 results. These subsets were equally distributed across stimulus conditions and counterbalanced across the subject group. Apart from the particular word forms presented in the assessment, all the test parameters (number and sequence of tasks, instructions, speed of presentation, etc.) were the same on both days:

- In the *Free Recall Task,* each participant had to reproduce as many new word forms as they could remember by typing them onto a prepared spreadsheet. The instruction was: “Please type in all the new words that you can remember below”.The response time was not limited.
- *Free-form Definition Task* was used to estimate the acquisition of the newly learned concept’s meaning and its correspondence with the surface form. Participants were given a table with a list of 10 newly learned items and the following instruction: “Below is the list of 10 new words you have encountered during the experiment. Try to define each one of them”.
- *Semantic Judgement Task (SJT)* was aimed at assessing the acquisition of novel words through making links between the newly learned word forms and their explicit definitions. Participants received the following instruction for this task: “You will be presented with a word and three definitions. You should choose one correct definition for each word by pressing the key labeled 1, 2, 3, or 4 on the response pad. The definition number 1 corresponds to key 1, the definition number 2—to key 2, number 3—to key 3, the option ‘None of this’—to key 4”. Only one of the definitions was correct, and two others were foils corresponding to other pseudowords. This task was implemented in NBS Presentation 20.0 with the same screen and text parameters as in the contextual acquisition part. The time to make the selection was not limited.

The order of the tasks was chosen to minimise any carry-over effects from one task to the following ones. Thus, all 3 tasks provided the assessment of the acquisition of both novel word forms and their semantics. The average time of the assessment task implementation was 20 min on each testing day. As already mentioned, the pairing between particular word forms and semantics was counterbalanced across participants, ruling out any idiosyncratic effects that could potentially arise if coupling specific word forms always with the same semantics. That implies that we could assess the lexical level (through recall) and the semantic level of meaning comprehension (via free-form definition and semantic judgement tasks) relatively independently.

### Data analysis

The results for concrete and abstract items were analysed on Day 1 and Day 2 separately. Both subject-(F1) and item-(F2) based analyses were implemented in order to assess any effects of or interactions between the factors.

In the free recall task, for the Day 2 only those recall results were analysed that had not been examined on Day 1 in the other two tasks, in order to avoid confounds related to reinforcement through repeated exposure to the same stimuli. To quantify these responses, we counted the number of correctly spelt letters in each word (thus giving a maximum of 8 points per correctly remembered word), and then converted them to percentage of correct responses in relation to the maximum possible.

Free-form definition task was assessed for two parameters: overall accuracy (the number of correct answers, i.e. those generally correctly matching the word form and its meaning irrespective of the amount of detail) and definition quality (completeness/quality of meaning description), which were evaluated in a quantitative fashion by four experts, who rated the definitions independently on a scale from 0 (definition does not suit any of the word’s features) to 5 (complete and accurate definition; see Supplementary materials, Table 2). The coherence of their assessment was handled with W-Kendall coefficient (which ranged between 0.791 and 0.856 across the participants) and average ratings were used for assessing the results statistically.

Semantic judgement task results were analysed in terms of accuracy (total number of correct responses expressed in percentage).

Three factors were used for statistical analysis: Testing Day (Day 1/Day 2), Semantic Type (Concrete/Abstract) and Stimulation Group (Sham/Anode/Cathode). To assess possible interactions between the three factors, repeated-measures Analysis of Variance (ANOVA) was used. The comparisons between days (to test putative consolidation effects) and between semantic types (concrete and abstract concepts for all tasks) on each day were carried out by within-group analyses using Wilcoxon sign-rank test for two related samples. To compare accuracies between groups (sham/cathodal tDCS, sham/anodal tDCS, anodal/cathodal tDCS) Wilcoxon rank-sum test for two independent samples was used. Multiple comparisons were corrected for using false discovery rate (FDR) algorithm^57^.

## Results

Below, we report results of within- and across-group analyses, grouped by the assessment task. To account for different measures across the tasks, they were normalised by converting to percentage of correct responses.

### Free recall

**Figure 2:**
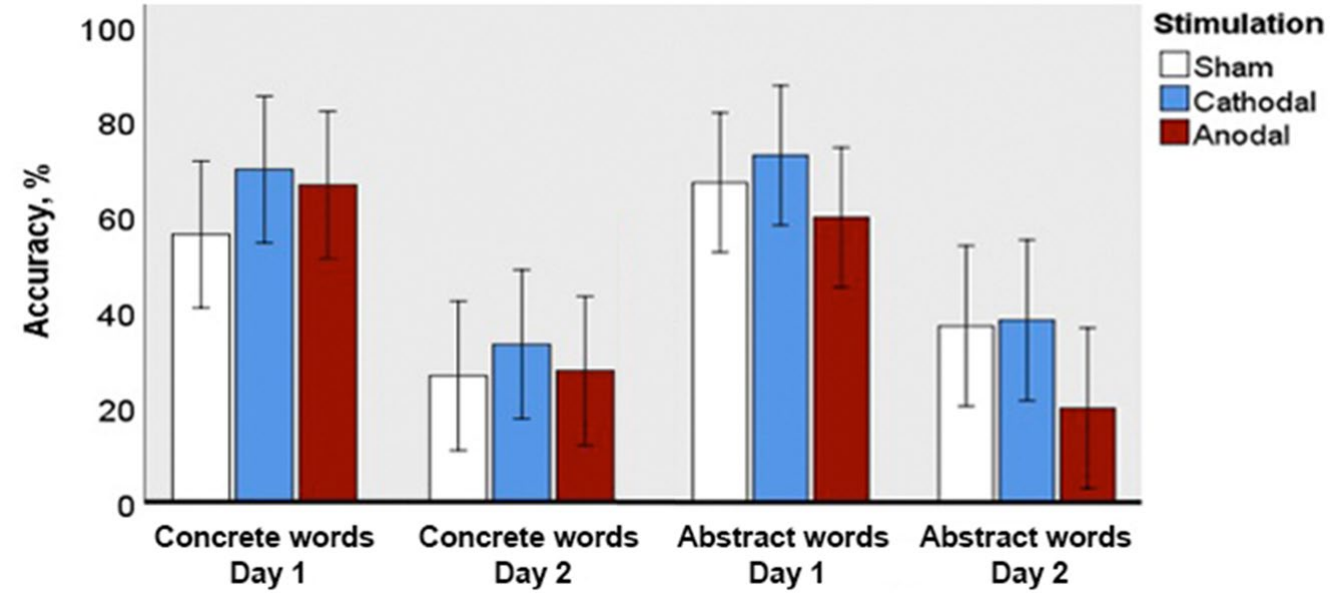
Accuracy in the free recall task for novel concrete and abstract words immediately after learning (Day 1) and following an overnight sleep (Day 2). Error bars denote 95% confidence intervals.

The free recall task (Fig. 2) indicated the ability of subjects to partially recall the acquired word forms even without any cues. In this task, there were neither within-group differences between target item types nor, in spite of visually observed numerical differences, significant between-group main effects. All general ANOVA results (including non-significant interactions) are reported in Supplementary materials file, table 3. Between-day analysis, however, revealed that accuracy for new concrete and abstract words on the first day was significantly higher than on the second day for sham (p = 0.006, Z = 2.9 for concrete and p = 0.006, Z = 3.1 for abstract words) and anodal (p = 0.006, Z = 3.0 for concrete and p = 0.006, Z = 3.0 for abstract words) stimulation. At the same time, in the cathodal group, the performance drop between days of assessment was not significant for the novel words of either of two types (p = 0.690, Z = 2.2 for concrete and p = 0.171, Z = 1.7 for abstract words). F1 ANOVA revealed significant main effect of Day (F(1;69) = 93.364; p = 0.0001; ηp^2^ = 0.576), confirmed by F2 ANOVA (F(1;29) = 86.603; p = 0.0001; ηp^2^ = 0.749) and reflecting the overall drop in performance between days.

### Free-form definition

**Figure 3:**
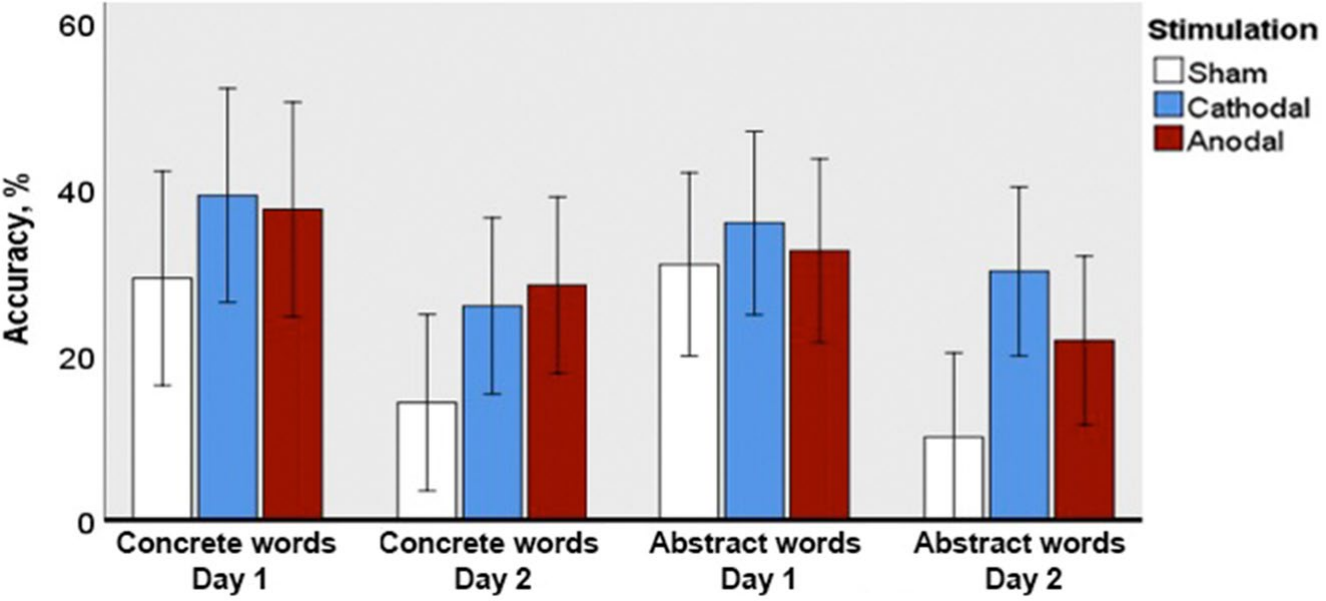
Accuracy in the free-form definition task for novel concrete and abstract words immediately after learning (Day 1) and following an overnight sleep (Day 2). Error bars denote 95% confidence intervals.

**Figure 4:**
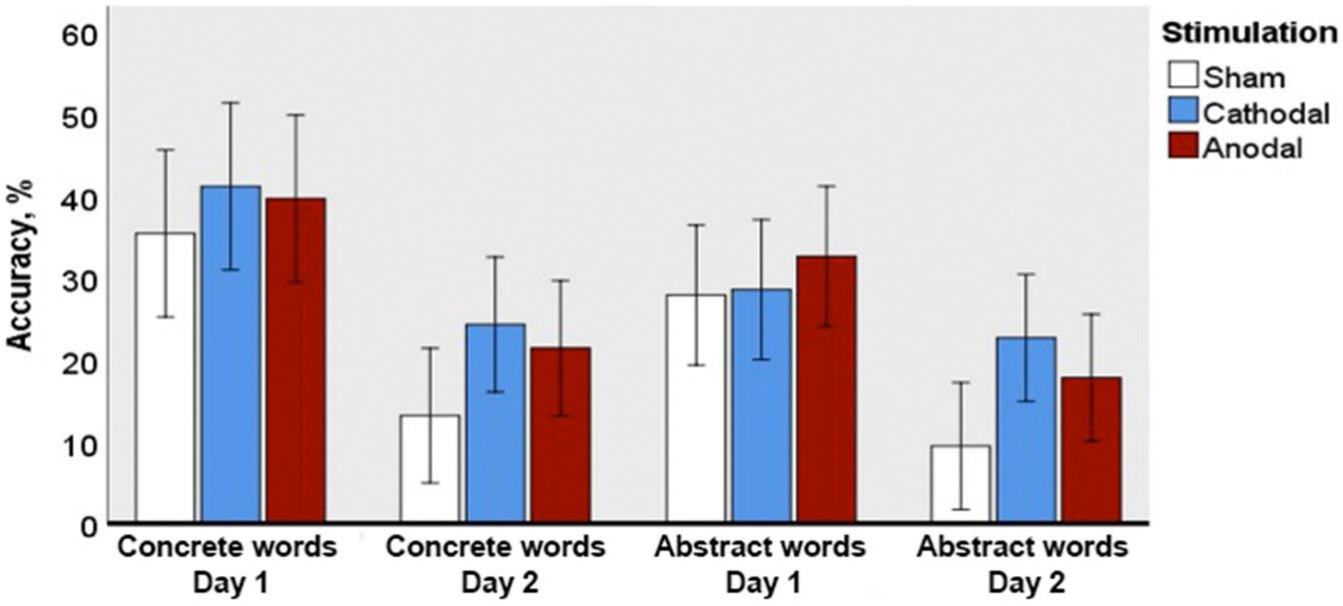
Definition quality ratings (%) in the free-form definition task for novel concrete and abstract words immediately after learning (Day 1) and following an overnight sleep (Day 2). Error bars denote 95% confidence intervals.

In the free-form definition task, two parameters were analysed: definition accuracy (Fig. 3) and definition quality (Fig. 4). Three-way F1 ANOVA (with factors Day, Group, and Type) revealed the main effect of Day (F(1; 69) = 38.165; p = 0.0001; ηp^2^ = 0.356) for *definition accuracy*, although the interaction between the factors did not reach significance.

Post-hoc Day 2 vs. Day 1 comparisons distinguished between the stimulation conditions. Particularly, they showed that accuracy performance decreased from Day 1 to Day 2 for abstract concepts (p = 0.006, Z = 3.1) in the sham group and, marginally, in the anodal (p = 0.069, Z = 2.0) groups, whereas in the cathodal group this decrease did not take place (p = 0.460, Z = 1.578).

F2 ANOVA confirmed the effect of the factor Day (F(1; 29) = 23.032; p = 0.0001; ηp^2^ = 0.443) and also revealed the effect of the Group (F(2; 58) = 8.845; p = 0.001; ηp^2^ = 0.234), which is driven by the generally better performance in real vs. sham stimulation conditions.

Implemented for *definition quality*, F1 ANOVA demonstrated significant effect of Day (F(1; 69) = 99.142; p = 0.0001; ηp^2^ 0.590) and stimulus Type (F(1; 69) = 16.919; p = 0.0001; ηp^2^ = 0.197) as well as an interaction at tendency level between Day and stimulus Type (F(1; 69) = 3.019; p = 0.087; ηp^2^ = 0.042) and Day and Group (F(2; 69) = 2.808; p = 0.067; ηp^2^ = 0.075). ANOVA for each testing day revealed the effect of stimulus Type on the first (F(1; 69) = 12.777; p = 0.001; ηp^2^ = 0.156) as well as on the second (F(1; 69) = 5.551; p = 0.021; ηp^2^ = 0.074) day of assessment, and no other interactions between factors on either of the days. Between-group effect was marginal on Day 2 (F(2; 69) = 2.991; p = 0.057; ηp^2^ = 0.080). This was confirmed by a post-hoc between-group analysis, which showed a significant better performance of the cathodal over the sham group for the abstract concepts on Day 2 (p = 0.038, Z = 2.2).

A post hoc comparison between Days 1 and 2 revealed an overnight drop in performance for both concrete and abstract concepts in the sham (p = 0.0001, Z = 4.0 for concrete and p = 0.000, Z = 3.8 for abstract semantics) and in the anodal (p = 0.0001, Z = 3.0 for concrete and p = 0.004, Z = 3.5 for abstract semantics) groups at significant levels as well as a significantly decreased performance of concrete (p = 0.0001, Z = 3.6) and only marginally—of abstract semantics (p = 0.06, Z = 2.2)—in the cathodal group. A post hoc comparison between semantic types in the free-form definition task revealed a better definition quality for concrete than abstract concepts on the first day in the cathodal group (p = 0.008, Z = 2.9) and on the second day in the anodal group (p = 0.015, Z = 2.4).

F2 ANOVA confirmed the main effects of Day (F(1; 29) = 56.786; p = 0.0001; ηp^2^ = 0.662), Type (F(1; 29) = 6.910; p = 0.014; ηp^2^ 0.443) and Group (F(2; 58) = 7.849; p = 0.001; ηp^2^ = 0.192), with no interactions between factors.

### Multiple-choice semantic judgement

**Figure 5:**
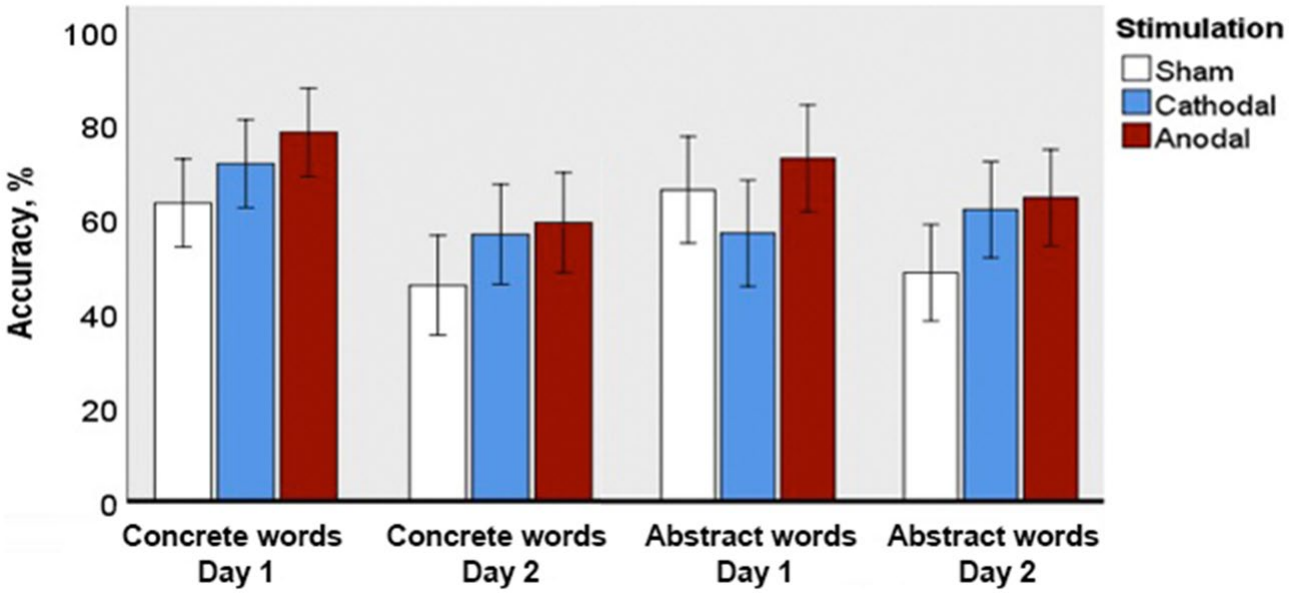
Accuracy in the semantic judgement task for novel concrete and abstract words immediately after learning (Day 1) and following an overnight sleep (Day 2). Error bars denote 95% confidence intervals.

F1 ANOVA revealed significant main effect of Day (F(1; 69) = 28.321; p = 0.0001; ηp^2^ = 0.291), as well as interactions between Day and Type (F(1; 69) = 5.513; p = 0.022; ηp^2^ = 0.074) and Day and Group, at tendency level (F(2; 69) = 2.660; p = 0.077; ηp^2^ = 0.072). Following this up with two separate analysis for each assessment day, we found the main effect of stimulation on the second (but not the first) day of assessment at the level of near-significant tendency (F (2; 69) = 2.944; p = 0.059; ηp^2^ = 0.079), with higher accuracy for abstract words in anodal tDCS than in sham group, albeit only at tendency level (p = 0.091; Z = − 2.2) (Fig. 5).

Within-group analysis revealed significant differences in accuracy between the two days of assessment. Particularly, a decreased performance on Day 2 in comparison with Day 1 was found for abstract (p = 0.015, Z = 2.7) and concrete (p = 0.032, Z = 2.4) semantics in the sham group and for concrete semantics in both anodal (p = 0.003, Z = 3.2) and cathodal (p = 0.044; Z = − 2.3) groups. A direct comparison between the semantic types revealed concreteness effect on Day 1 for the cathodal group only (p = 0.044; Z = − 2.2).

F2 ANOVA affirmed the effect of Day (F(1;29) = 19.632; p = 0.0001; ηp^2^ = 0.404) and interactions between Day and Group (F(2;58) = 2.848; p = 0.066; ηp^2^ = 0.089). It also revealed a significant main effect of Group (F(2;58) = 8.728; p = 0.0001; ηp^2^ = 0.231).

## Discussion

The present study is, to our knowledge, one of the first to apply the tDCS method to modulate language learning, particularly the acquisition of concrete vs. abstract semantics. The findings of the study demonstrate successful acquisition of both concrete and abstract words following just 5 presentations in sentential context in what was a rather challenging task of learning 20 new words in a single reading session. Moreover, the overall learning trajectory seemed to differ between the sham group and the two stimulation groups. We ran a number of tests (FDR-corrected for multiple comparisons) to check the word acquisition outcomes and found the effects of tDCS in all assessment tasks. Whereas all tests showed an overall reduction in performance between Days 1 and 2 (not surprising considering the complexity of the task and the number and diversity of words to be acquired), the effects on both days and this change in performance differed between the three stimulation groups and the linguistic stimulus types.

The free recall task, whose main purpose was to assess the acquisition of word forms per se, irrespective of their semantics, showed a limited amount of effects. The most interesting outcome was that the drop in recall hits on Day 2, whilst being significant in both sham and anodal tDCS groups for both concrete and abstract stimuli, was not significant in the cathodal tDCS group, which potentially might suggest a positive effect of cathodal Wernicke tDCS on consolidation of long-term memory traces for new word forms. Note that this tentative interpretation is based on the main effects; as it was not supported by significant interaction between factors in the ANOVA, it must be treated with extreme caution, and perhaps a larger sample is needed to test this conjecture. However, similar results were observed for definition accuracy and multiple-choice semantic judgment tasks (discussed below), which indirectly supports this hypothesis.

Interestingly, this lexical-level test did not differentiate the learning performance between novel concrete and abstract words, indicating overall similar acquisition of both types of word forms. This lack of semantically-specific effects was confirmed by both F1 (conventional subject-based) and F2 (item-based) analyses. This is different from the well-known “concreteness effect”, and may be explained by the highly controlled nature of the paradigm, in which both sets of words were fully matched for surface properties through counterbalancing, and were learned in an identical fashion through highly similar short-story contexts. Of all tests applied, only the analysis of definition quality led to limited concreteness effects; considering that the definition accuracy did not show a similar divergence, this may have more to do with the nature of assessment task and the relative ease of defining concrete objects, than with poorer learning as such. This suggests that the conventional concreteness effect may, at least in part, possibly be driven by different psycholinguistic properties (e.g. length, lexical frequency), age of acquisition, context of use, and other variables, rather than the concreteness/abstractness of words per se.

Both semantic tasks—free-form definition and multiple-choice semantic judgement—indicated further differences between the three groups, with an overall better performance of both tDCS groups over the sham one. Furthermore, following corrections for multiple comparisons, this effect approached significance on Day 2 for abstract semantics in both tasks, although diverging between stimulation polarities. These tasks specifically tested the ability to assess and describe the features of the novel concepts, before and after their putative binding into a memory circuit during the overnight consolidation phase^58,59^, the stage that seemed to be positively affected by stimulation. When looking at individual word types, the free-form definition task also indicated better accuracy for abstract semantics on Day 2 for the cathodal group, as opposed to sham, whereas a similar effect for the anodal group was not found. This diverse effect of the stimulation polarity is line with the existing literature which indicated differential activation for pre-existing concrete and abstract words in posterior temporal and inferior parietal structures in the Wernicke area vicinity^60–62^. Based on existing lesion studies, it is known that left temporoparietal cortex plays a crucial role in abstract words processing^63^.

Furthermore, whereas a drop in the definition quality performance from Day 1 to Day 2 was fully significant for the abstract items in both sham and anodal groups, it did not reach full significance in the cathodal one. In similar vein, SJT showed a significant between-day drop in accuracy for abstract and concrete words in the sham group as well as for concrete semantics in the anodal one, but no significant reduction for the cathodal group. These two effects are highly similar to the overall better overnight preservation of performance in the free recall task above, and again suggest a positive effect of cathodal Wernicke tDCS on consolidation of new word memory circuits. At the semantic level, however, this effect seems to somewhat favour the consolidation of abstract words, indicating an important role of Wernicke’s area in their acquisition, consolidation, and storage. This may also be related to the putative importance of core language areas in storing abstract representations^16,62,64–66^, whereas concrete words may also rely on referentially specific sensory and motor modal areas, diminishing the relative input of core language cortex in their representations^14,64,67^. Thus, these results suggest overlapping but still distinct memory circuits underpinning encoding and storage of these two types of semantics, indicating a relatively more important role of the core language systems in maintaining amodal abstract representations. Our findings along with previous neuroimaging studies of concrete and abstract word processing^11–15^ therefore support the extended dual-coding approach, which explains the differences between abstract and concrete representations by positing their differential dependence on verbal-based and imagery-based (non-verbal) semantic memory systems. Although interrelated, they are recruited to different degrees, with the verbal system involved in coding both concrete and abstract concepts linguistically, while the imagery system is primarily involved in coding concrete, but not abstract concepts^8,11^, thereby putting a heavier load on the linguistic system—and thus the core language areas of the left hemisphere—in storing abstract knowledge. This framework would also predict a similar divergence between concrete and abstract word acquisition for stimulation of other key parts of the language system, such as Broca’s area and anterior-temporal lobe (ATL/temporal pole), which should be tested in future studies.

The present data corroborate previous results, which showed that the application of anodal tDCS over Wernicke’s area while learning new words significantly improved the accuracy and decreased latencies in a picture-naming task^34^, whereas anodal tDCS over posterior left perisylvian areas was demonstrated to positively affect associative verbal learning^28^. However, unlike previous studies, we have also attempted to compare the acquisition of abstract and concrete semantics in a controlled fashion, finding subtle differences between these processes as detailed above.

Although we find this putatively better expressed word learning advantage for cathodal stimulation over the sham condition, the direct comparison between the anodal and cathodal tDCS groups did not produce clear statistical effects. Numerically, both conditions indicated better performance over the sham one, even though, likely due to variance, the anodal stimulation led to statistically less clear improvements in learning outcomes. This is in contrast to what is known from tDCS studies of the motor system, which indicated inhibitory effects of cathodal tDCS and excitatory influence of anodal stimulation on motor performance and learning^39,68,69^. This suggests that the polarity effects, known from motor system stimulation, are not universal and may instead be specific to particular neurocognitive functions, which, in turn, is most likely related to the cortical geometry (e.g. curvature, gyrification, density, and other tissue properties, affecting the spread of currents) as well as possibly other (e.g. cytochemical) properties of the area stimulated. Indeed, whereas previous studies have confirmed excitatory effects (higher accuracy and/or decreased latencies) of anodal tDCS on verbal fluency^39^, picture-naming task^34^, working memory^70^, associative verbal learning^28^, semantic retrieval^71^, language comprehension^40^, there was no inhibitory effect of cathodal tDCS observed in those studies. This, as mentioned above, may be linked to specific tissue properties as well as to the balance of inhibition and excitation, where inhibiting some neurons may in fact lead to *dis*inhibiting certain functional circuits, making them more plastic in the case of a learning study.

Together with some of the previous studies, our findings may suggest a general effect of stimulation rather than the effect of a certain polarity. One explanation of the more general polarity-independent effect could be tDCS interference with or upregulating influence on the arousal and attention levels, possibly related to its ability to affect a variety of cognitive brain functions (Reinhart et al., 2017), although, notably, this would also apply in case of previous motor studies. It is also possible that the change in the activity of neurons during the stimulation affects the post-stimulation balance between the signal and noise that the brain is constantly trying to maintain. Ultimately, as it seems, this issue of polarity can only be satisfactorily addressed in wet in vivo or in vitro tissue studies, and we can only speculate about its possible underlying mechanisms in such non-invasive human experiments as the present one.

Crucially, we found the effects of tDCS not only and not so much in the immediate learning outcomes after the learning session, but also on a longer-term scale, after a 24-h period including an overnight sleep. It has been suggested that sleep may play an important role in word acquisition and other types of learning^59^ possibly due to its key role in memory consolidation and transfer of new traces from short-term to long-term memory^58^. Sleep appears to facilitate memory for abstract relations of words of an artificial language in infants ^72^ and benefits the integration of newly learned words into pre-existing knowledge in both school children and adults^73,74^. The longer-term effects of tDCS on language learning shown for the first time here may putatively suggest its potential applicability as a non-invasive assistive technology for ameliorating developmental learning deficits and acquired language disorders and possibly even as an educational aid. However, it goes without saying that substantial research will be needed to assess its usability and safety for such purposes.

Whereas we aimed at targeting functionally specific brain area, caution should be still applied with respect to neuroanatomical precision of tDCS. Unlike direct cortical stimulation or TMS, the precision of tDCS is generally considered rather low and thus we cannot assume focal stimulation of Wernicke's area only. On the positive side, the current distribution model calculated for our stimulation settings clearly indicated that most of the stimulation effect was concentrated temporo-parietally, with maxima within Wernicke’s area. Still, this, on the one hand, does not rule out stimulation of other areas which the current passes through, and, on the other hand, does not guarantee complete stimulation of the target zone with even intensity throughout. This is, however, a general feature of this technique, which, on the more positive side, is well compensated by its safety, non-invasiveness, ease of use, and low cost. Future studies may use a more focal stimulation technique, such as TMS, to verify the present results. Yet another way to control the outcomes in future neuromodulation studies could be to use double-blind experimental designs^75,76^.

On another cautious note, the present study used a between-group design, which is open to criticisms with respect to its lower statistical sensitivity as well as possible confounds related to potential baseline group differences. The choice of between-group design in the study was determined by the learning paradigm, which made it impossible to test the same subjects three times in the same learning task. That said, the critical findings were produced here by within-group contrasts, comparing stimuli and days of assessment within each group. At the same time, the groups were matched by gender, age, handedness, the experience of foreign language learning, and the level of education, largely ruling out any baseline group differences as the explanation of the effects found. However, future studies could still attempt to modify this paradigm and use a within-subject design to overcome the drawbacks pertinent to group comparisons.

In sum, the present results suggest that Wernicke’s area direct current stimulation may improve contextual acquisition of new words at both lexical and semantic levels generally, and of novel concrete and abstract semantics specifically. Furthermore, they demonstrate a somewhat more robust effect of cathodal stimulation on learning in general and on the acquisition of novel abstract knowledge in particular, indicating overlapping but diverging brain mechanisms for concrete and abstract semantics, and highlighting a possibly more important role of core language areas in storing abstract knowledge. The results of this study, while they may potentially pave the way for future neuroassistive technologies, should still be treated with caution, and must be verified and extended in future studies. Further investigations could use different learning paradigms (for instance, explicit encoding, fast-mapping, word-picture association, visual and auditory inputs, etc.), stimulation regimes (e.g. duration, intensity, and shape of the current), brain areas (e.g. Broca’s area, left anterior-temporal pole, their right-hemispheric homologues, etc.), and experimental languages to fully elucidate the potential of tDCS for modulating language learning and the role of different brain areas in acquiring different types of semantics.

## Supplementary Information

Supplementary Information

## References

[1] Borghi AM, Binkofski F, Borghi A, Binkofski F (2014). The problem of definition. Words as Social Tools: An Embodied View on Abstract Concepts.

[2] Jessen F (2000). The concreteness effect: evidence for dual coding and context availability. Brain Lang..

[3] Pexman PM, Hargreaves IS, Edwards JD, Henry LC, Goodyear BG (2007). Neural correlates of concreteness in semantic categorization. J. Cogn. Neurosci..

[4] Schwanenflugel PJ, Akin C, Luh WM (1992). Context availability and the recall of abstract and concrete words. Mem. Cognit..

[5] Fliessbach K, Weis S, Klaver P, Elger CE, Weber B (2006). The effect of word concreteness on recognition memory. Neuroimage.

[6] Schwanenflugel PJ, Shoben EJ (1983). Differential context effects in the comprehension of abstract and concrete verbal materials. J. Exp. Psychol. Learn. Mem. Cogn..

[7] Mestres-Missé A, Münte TF, Rodriguez-Fornells A (2014). Mapping concrete and abstract meanings to new words using verbal contexts. Second Lang. Res..

[8] Paivio A (1990). Mental representations: a dual coding approach.

[9] Crutch SJ (2006). Qualitatively different semantic representations for abstract and concrete words: Further evidence from the semantic reading errors of deep dyslexic patients. Neurocase..

[10] Mkrtychian N (2019). Concrete vs abstract semantics: From mental representations to functional brain mapping. Front. Hum. Neurosci..

[11] Holcomb PJ, Kounios J, Anderson JE, West WC (1999). Dual-coding, context-availability, and concreteness effects in sentence comprehension: an electrophysiological investigation. J. Exp. Psychol. Learn. Mem. Cogn..

[12] Khachatryan E, Hnazaee MF, Van Hulle MM (2018). Effect of word association on linguistic event-related potentials in moderately to mildly constraining sentences. Sci. Rep..

[13] Della Rosa PA, Catricalà E, Canini M, Vigliocco G, Cappa SF (2018). The left inferior frontal gyrus: A neural crossroads between abstract and concrete knowledge. Neuroimage..

[14] Moseley RL, Pulvermüller F (2014). Nouns, verbs, objects, actions, and abstractions: Local fMRI activity indexes semantics, not lexical categories. Brain Lang..

[15] Dreyer FR, Pulvermüller F (2018). Abstract semantics in the motor system?—An event-related fMRI study on passive reading of semantic word categories carrying abstract emotional and mental meaning. Cortex..

[16] Binder JR, Westbury CF, McKiernan KA, Possing ET, Medler DA (2005). Distinct brain systems for processing concrete and abstract concepts. J. Cogn. Neurosci..

[17] Anzellotti S, Caramazza A, Saxe R (2017). Multivariate pattern dependence. PLoS Comput. Biol..

[18] Manenti R, Brambilla M, Petesi M, Ferrari C, Cotelli M (2013). Enhancing verbal episodic memory in older and young subjects after non-invasive brain stimulation. Front. Aging Neurosci..

[19] Vukovic N, Feurra M, Shpektor A, Myachykov A, Shtyrov Y (2017). Primary motor cortex functionally contributes to language comprehension: An online rTMS study. Neuropsychologia..

[20] Scorolli C (2012). Abstract and concrete phrases processing differentially modulates cortico-spinal excitability. Brain Res..

[21] Hoffman P, Jefferies E, Lambon Ralph MA (2010). Ventrolateral prefrontal cortex plays an executive regulation role in comprehension of abstract words: Convergent neuropsychological and repetitive TMS evidence. J. Neurosci..

[22] Orena EF, Caldiroli D, Acerbi F, Barazzetta I, Papagno C (2019). Investigating the functional neuroanatomy of concrete and abstract word processing through direct electric stimulation (DES) during awake surgery. Cogn. Neuropsychol..

[23] Reilly J, Kean J (2007). Formal distinctiveness of high- and low-imageability nouns: Analyses and theoretical implications. Cogn. Sci..

[24] Vigliocco G, Ponari M, Norbury C (2018). Learning and processing abstract words and concepts: Insights from typical and atypical development. Top. Cogn. Sci..

[25] Glanzer M, Bowles N (1976). Analysis of the word-frequency effect in recognition memory. J. Exp. Psychol. Hum. Learn. Mem..

[26] Paivio A, Yuille JC, Madigan SA (1968). Concreteness, imagery, and meaningfulness values for 925 nouns. J. Exp. Psychol..

[27] Brysbaert M, Mandera P, McCormick SF, Keuleers E (2019). Word prevalence norms for 62,000 English lemmas. Behav. Res. Methods..

[28] Flöel A, Rösser N, Michka O, Knecht S, Breitenstein C (2008). Noninvasive brain stimulation improves language learning. J. Cogn. Neurosci..

[29] Branscheidt M, Hoppe J, Freundlieb N, Zwitserlood P, Liuzzi G (2017). tDCS over the motor cortex shows differential effects on action and object words in associative word learning in healthy aging. Front. Aging Neurosci..

[30] Hartwigsen G (2015). Modeling the effects of noninvasive transcranial brain stimulation at the biophysical, network, and cognitive level. Progr. Brain Res..

[31] Flöel A (2012). Non-invasive brain stimulation improves object-location learning in the elderly. Neurobiol. Aging..

[32] Zimerman M (2013). Neuroenhancement of the aging brain: Restoring skill acquisition in old subjects. Ann. Neurol..

[33] Blagovechtchenski E (2019). Transcranial direct current stimulation (tDCS) of Wernicke’s and Broca’s areas in studies of language learning and word acquisition. J. Vis. Exp..

[34] Fiori V (2011). Transcranial direct current stimulation improves word retrieval in healthy and nonfluent aphasic subjects. J. Cogn. Neurosci..

[35] Pulvermüller F (2013). How neurons make meaning: Brain mechanisms for embodied and abstract-symbolic semantics. Trends Cognit. Sci..

[36] Schmidt S, Fleischmann R, Bathe-Peters R, Irlbacher K, Brandt SA (2013). Evolution of premotor cortical excitability after cathodal inhibition of the primary motor cortex: A sham-controlled serial navigated TMS study. PLoS ONE.

[37] Pellicciari MC, Brignani D, Miniussi C (2013). Excitability modulation of the motor system induced by transcranial direct current stimulation: A multimodal approach. Neuroimage..

[38] Bastani A, Jaberzadeh S (2013). A-tDCS differential modulation of corticospinal excitability: The effects of electrode size. Brain Stimul..

[39] Iyer MB (2005). Safety and cognitive effect of frontal DC brain polarization in healthy individuals. Neurology..

[40] Sparing R, Dafotakis M, Meister IG, Thirugnanasambandam N, Fink GR (2008). Enhancing language performance with non-invasive brain stimulation—A transcranial direct current stimulation study in healthy humans. Neuropsychologia..

[41] Rogalewski A, Breitenstein C, Nitsche MA, Paulus W, Knecht S (2004). Transcranial direct current stimulation disrupts tactile perception. Eur. J. Neurosci..

[42] Berryhill ME, Wencil EB, Branch Coslett H, Olson IR (2010). A selective working memory impairment after transcranial direct current stimulation to the right parietal lobe. Neurosci. Lett..

[43] Weiss M, Lavidor M (2012). When less is more: Evidence for a facilitative cathodal tDCS effect in attentional abilities. J. Cogn. Neurosci..

[44] Pirulli C, Fertonani A, Miniussi C (2014). Is neural hyperpolarization by cathodal stimulation always detrimental at the behavioral level?. Front. Behav. Neurosci..

[45] Brückner S, Kammer T (2017). Both anodal and cathodal transcranial direct current stimulation improves semantic processing. Neuroscience.

[46] Nitsche MA (2003). Modulation of cortical excitability by weak direct current stimulation—technical, safety and functional aspects. Suppl. Clin. Neurophysiol..

[47] Priori A (2003). Brain polarization in humans: A reappraisal of an old tool for prolonged non-invasive modulation of brain excitability. Clin. Neurophysiol..

[48] Shah PP, Szaflarski JP, Allendorfer J, Hamilton RH (2013). Induction of neuroplasticity and recovery in post-stroke aphasia by non-invasive brain stimulation. Front. Hum. Neurosci..

[49] Dumay N, Gaskell MG (2012). Overnight lexical consolidation revealed by speech segmentation. Cognition.

[50] Bastani A, Jaberzadeh S (2012). Does anodal transcranial direct current stimulation enhance excitability of the motor cortex and motor function in healthy individuals and subjects with stroke: A systematic review and meta-analysis. Clin. Neurophysiol..

[51] Binder JR, Desai RH, Graves WW, Conant LL (2009). Where is the semantic system? A critical review and meta-analysis of 120 functional neuroimaging studies. Cereb. Cortex..

[52] Oldfield RC (1971). The assessment and analysis of handedness: The Edinburgh inventory. Neuropsychologia..

[53] Thair H, Holloway AL, Newport R, Smith AD (2017). Transcranial direct current stimulation (tDCS): A Beginner’s guide for design and implementation. Front. Neurosci..

[54] Nitsche MA (2007). Shaping the effects of transcranial direct current stimulation of the human motor cortex. J. Neurophysiol..

[55] Saturnino GB, Makarov S, Horner M, Noetscher G (2019). SimNIBS 2.1: A comprehensive pipeline for individualized electric field modelling for transcranial brain stimulation. Brain and Human Body Modeling.

[56] Kessler SK, Turkeltaub PE, Benson JG, Hamilton RH (2012). Differences in the experience of active and sham transcranial direct current stimulation. Brain Stimul..

[57] Hochberg Y, Benjamini Y (1990). More powerful procedures for multiple significance testing. Stat. Med..

[58] Rasch B, Born J (2013). About sleep’s role in memory. Physiol. Rev..

[59] Davis MH, Gaskell MG (2009). A complementary systems account of word learning: Neural and behavioural evidence. Philos. Trans. R. Soc. B Biol. Sci..

[60] Binder JR (2015). The Wernicke area: Modern evidence and a reinterpretation. Neurology..

[61] Hoffman P, Binney RJ, Lambon Ralph MA (2015). Differing contributions of inferior prefrontal and anterior temporal cortex to concrete and abstract conceptual knowledge. Cortex..

[62] Papagno C, Martello G, Mattavelli G (2013). The neural correlates of abstract and concrete words: Evidence from brain-damaged patients. Brain Sci..

[63] Skipper-Kallal LM, Mirman D, Olson IR (2015). Converging evidence from fMRI and aphasia that the left temporoparietal cortex has an essential role in representing abstract semantic knowledge. Cortex..

[64] Mårtensson F, Roll M, Apt P, Horne M (2011). Modeling the meaning of words: Neural correlates of abstract and concrete noun processing. Acta Neurobiol. Exp. (Wars).

[65] Roll M (2012). Atypical associations to abstract words in Broca’s aphasia. Cortex..

[66] Sabsevitz DS, Medler DA, Seidenberg M, Binder JR (2005). Modulation of the semantic system by word imageability. Neuroimage..

[67] Humphreys GW, Riddoch MJ, Price CJ (1997). Top-down processes in object identification: Evidence from experimental psychology, neuropsychology and functional anatomy. Philos. Trans. R. Soc. B Biol. Sci..

[68] Cattaneo Z, Pisoni A, Papagno C (2011). Transcranial direct current stimulation over Broca’s region improves phonemic and semantic fluency in healthy individuals. Neuroscience.

[69] Fertonani A, Rosini S, Cotelli M, Rossini PM, Miniussi C (2010). Naming facilitation induced by transcranial direct current stimulation. Behav. Brain Res..

[70] Brunoni AR (2012). Clinical research with transcranial direct current stimulation (tDCS): Challenges and future directions. Brain Stimul..

[71] Ihara AS (2015). Facilitated lexical ambiguity processing by transcranial direct current stimulation over the left inferior frontal cortex. J. Cogn. Neurosci..

[72] Gómez RL, Bootzin RR, Nadel L (2006). Naps promote abstraction in language-learning infants. Psychol. Sci..

[73] Dumay N, Gaskell MG (2007). Sleep-associated changes in the mental representation of spoken words: Research report. Psychol. Sci..

[74] Henderson LM, Weighall AR, Brown H, Gaskell MG (2012). Consolidation of vocabulary is associated with sleep in children. Dev. Sci..

[75] Brunoni AR, Boggio PS, Ferrucci R, Priori A, Fregni F (2013). Transcranial direct current stimulation: Challenges, opportunities, and impact on psychiatry and neurorehabilitation. Front. Psychiatry..

[76] Richardson JD (2014). Sham protocols for transcranial direct current stimulation using high-definition electrodes. Brain Stimul..

