# Supplementary material for: Acquisition of concrete and abstract words is modulated by tDCS of Wernicke’s area
Source: Sci Rep. 2021 Jan 15;11:1508. doi: 10.1038/s41598-020-79967-8 (PMC7811021; doi:10.1038/s41598-020-79967-8)
Supplement: Supplementary file 1 — Supplementary Information [file 41598_2020_79967_MOESM1_ESM.pdf]

Acquisition of concrete and abstract words modulated by tDCS of Wernicke's area

Diana Kurmakaeva[1,+,*], Evgeny Blagovechtchenski[1,+], Daria Gnedykh[1,+], Nadezhda Mkrtychian[1,+], Svetlana Kostromina[1,+], and Yury Shtyrov[1,2,+]

[1]Saint Petersburg State University, Laboratory of Behavioural Neurodynamics, Saint Petersburg, 199004, Russian Federation
[2]Center of Functionally Integrative Neuroscience (CFIN), Aarhus University, Department of Clinical Medicine,
Aarhus, 8000, Denmark
[*]diana.s-pb@mail.ru
[+]these authors contributed equally to this work

Чтобы снизить риск для жизни людей, используется циркунал.<br><br>[To minimize any risks for human lives, they use circunal.]
На выставке робототехники в Москве был представлен циркунал.<br><br>[At the robot exhibition in Moscow, you could find a circunal.]
Совсем скоро в арсенале пожарных может появиться циркунал.<br><br>[Very soon firefighters will be able to use the circunal.]
Иван вынес из огня ребенка, которого нашел циркунал.<br><br>[Ivan rushed out from the burning house carrying a child found by the circunal.]
Во время учений в прокладке шланга участвовал циркунал.<br><br>[During the fire training, the hose was being laid by a new circunal.]

Table 1. Example of contextual sentences presented during the learning session

Score	Description
0	Definition does not suit any of the word’s features.
1	The category of the concept is named (e.g., "some object" or "some feeling"), the concept is not defined;
2	Definition matches the semantic field (e.g. "smth related to cooking"), but the concept is not defined precisely.
3	The concept is defined partially, according to its formal features but the core features are indicated with errors or not indicated.
4	The concept is defined partially (without naming 1-2 formal features).
5	Complete and accurate definition of the concept with all its features.

Table 2. Assessment criteria of participants’ definitions suggested by them in Free-form definition task

Source	F	df	df (error)	Sig.	Partial Eta Squared
Free recall					
Group	1.011	2	69	0.369	0.028
Day	93.364	1	69	0.000	0.575
Type	0.276	1	69	0.599	0.004
Day*Group	0.632	2	69	0.535	0.018
Type*Group	0.399	2	69	0.254	0.039
Day*Type	0.002	1	69	0.963	0.000
Day*Group*Type	0.009	2	69	0.991	0.000
Free-form definition. Definition accuracy					
Group	1.903	2	69	0.157	0.052
Day	31.438	1	69	0.000	3.313
Type	0.911	1	69	0.343	0.013
Day*Group	1.479	2	69	0.235	0.041
Type*Group	0.644	2	69	0.528	0.018
Day*Type	0.000	1	69	1.000	0.000
Day*Group*Type	0.659	2	69	0.521	0.019
Free-form definition. Definition quality					
Group	1.267	2	69	0.288	0.035
Day	90.975	1	69	0.000	0.569
Type	13.723	1	69	0.000	0.166
Day*Group	2.377	2	69	0.100	0.064
Type*Group	0.115	2	69	0.891	0.003
Day*Type	4.173	1	69	0.045	0.057
Day*Group*Type	0.681	2	69	0.510	0.019
Multiple-choice semantic judgment					
Group	2.588	2	69	0.082	0.070
Day	28.321	1	69	0.000	0.291
Type	0.180	1	69	0.673	0.003
Day*Group	2.660	2	69	0.077	0.072
Type*Group	0.905	2	69	0.409	0.026
Day*Type	5.513	1	69	0.022	0.074
Day*Group*Type	1.744	2	69	0.182	0.048

Table 3. The results of the ANOVA
